# Breaching Pathogeographic Barriers by the Bat White-Nose Fungus

**DOI:** 10.1128/mBio.00897-18

**Published:** 2018-05-22

**Authors:** Johanna Rhodes, Matthew C. Fisher

**Affiliations:** a MRC Centre for Global Infectious Disease Analysis, Imperial College London, London, United Kingdom

**Keywords:** epizootic, genome analysis, microsatellite, Pseudogymnoascus destructans, whole-genome sequencing, wildlife disease, white-nose syndrome

## Abstract

Bat white-nose syndrome has become associated with unparalleled mortality in bat species across the United States since 2006. In a recent article, Drees and colleagues (mBio 8:e01941-17, 2017, https://doi.org/10.1128/mBio.01941-17) utilized both whole-genome sequencing and microsatellite data to explore the origin and spread of the causative agent of bat white-nose syndrome, Pseudogymnoascus destructans. The research by Drees et al. supports the hypothesis that P. destructans was introduced into North America from Europe, with molecular dating suggesting a divergence from European isolates approximately 100 years ago. The approaches described in this study are an important contribution toward pinpointing the origins of this infection and underscore the need for more rigorous international biosecurity in order to stem the tide of emerging fungal pathogens.

## COMMENTARY

**FIG 1:**
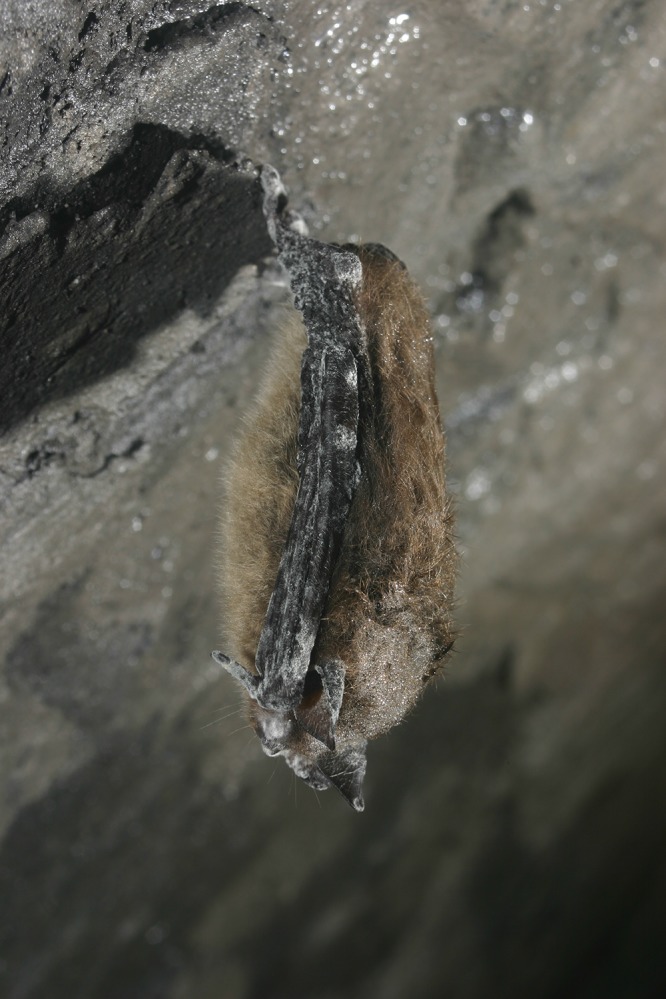
Little brown bat (Myotis lucifugus) infected by WNS. Photograph credit: A. Hicks, used with permission.

In their recent study, Drees and colleagues used whole-genome sequencing (WGS) of a large collection of bat white-nose fungus Pseudogymnoascus destructans isolates to identify three distinct populations of the fungus: high-diversity European isolates, which encompass the epizootic North American outbreak isolates, and two genetic outliers from China and Mongolia (1). Like all organisms, pathogens are constrained by geography resulting in global heterogeneities in their distributions, known as pathogeography. Increasingly however, globalization is eroding pathogeographic distributions through the action of long-distance movements of infectious propagules within global networks of travel and trade, seeding new outbreaks in new places and in naïve host species (2). Emerging fungal pathogens (EFPs) are becoming increasingly recognized as potent drivers of change as they are transported worldwide, damaging agriculture, colonizing new host species, and affecting communities of organisms inhabiting ecosystems worldwide (3, 4). Pertinently, in early 2006 a new disease named “white-nose syndrome” (WNS) was observed and documented in the Howes Cave bat hibernaculum west of Albany, NY, where it caused mass mortality in the little brown bat species inhabiting the cave (Fig. 1). The etiological agent of this fungal epizootic, characterized by the noticeable white fungal growth on muzzles and ear and wing membranes of infected bats (5), was identified as P. destructans (previously *Geomyces* spp.) (6). A rapid decline in bat populations followed the discovery of this fungus, with mortalities of ~6 million bats occurring across seven species in eastern North America (7), leading to the possibility of several species of bat facing local extinction. However, the pathogeographic origin of this aggressive EFP remained, until now, cryptic.

Initial studies based on microsatellite data provided insights into the spatial distribution and relationships between P. destructans isolates collected from across Europe and North America, suggesting a European origin of the infection (7, 8). WGS has the potential to provide much greater genetic resolution into patterns of gene flow within and between bat hibernacula and continents; the study by Drees et al. (1) adds to previous WGS completed by Trivedi et al. (9), confirming the spatial expansion of a highly clonal population from a single genotype that has begun to accumulate genetic variation via spontaneous mutation. There is yet no evidence of genetic exchange or recombination observed in North America owing to the occurrence in this region of only a single mating type of this sexual (heterothallic) fungus.

The newly sequenced North American isolates formed a monophyletic group, which were shown to be a nested subset of the genetic diversity of P. destructans present within Europe. Moreover, the European population of P. destructans was shown to be sexually recombining with both mating types present. That there is also an absence of mass mortality seen in European bats suggests P. destructans has coevolved with European bats well before a single outbreak genotype invaded North American bat hibernacula, triggering the mass mortalities. Dating analysis performed by Drees et al. suggests diversification of North American and European isolates within the last 100 years and divergence from Asian genotypes approximately 3,400 years ago. That the Chinese isolate appeared basal to isolates from Mongolia, Europe, and North America suggests Asia may contain the historical origin of P. destructans. It is assumed that bats can transport P. destructans over long distances, thus transporting P. destructans into Europe from western Asia and the Russian Federation (10). Asia has recently been identified as the origin of other EFPs, which have spread across the globe and subsequently diversified, including Batrachochytrium salamandrivorans (11), chestnut blight fungus Cryphonectria parasitica (12), and wheat yellow rust Puccinia striiformis f. sp. *tritici* (13). An Asian origin of P. destructans has been hypothesized by multiple studies (14, 15), and this hypothesis, along with the mode of dispersal and subsequent introduction into Europe and North America, needs to be investigated further.

Through the globalization of trade in forestry, agriculture and wildlife, there have been numerous accidental introductions of fungal pathogens into nonnative ecosystems. Well-studied examples include the amphibian-parasitizing chytrid B. salamandrivorans invading Europe from Asia (11) alongside its relative Batrachochytrium dendrobatidis, becoming globally ubiquitous; both appear to have invaded naïve ecosystems using the trade in amphibians as a bridgehead (16). Similarly, snakes captured in the wild and transported across the globe are now known to be infected with the causative agent of snake fungal disease (SFD), Ophidiomyces ophiodiicola (17, 18). Although currently not known, the transport of P. destructans may have occurred via the dispersal of contaminated soil from Europe into North America (19) as a consequence of outdoor pursuits, such as caving. If true, WGS now holds the exciting promise of identifying the exact cave from which this EFP stemmed.

Rapid genome sequencing is essential to gain a better understanding of the epidemiology and evolution of EFPs, including P. destructans. Drees et al. use genetic markers to provide information on the origin and spread of P. destructans. However, research efforts into other EFPs, such as the ash dieback pathogen Hymenoscyphus fraxineus, have identified markers indicative of disease tolerance in the host (20); future efforts at investigating WNS could focus on host-pathogen interactions to look for local patterns of diversification which may be indicative of rapid nature selection and local adaptation. To this end, Drees et al. reported two samples collected in Indiana that have formed a geographically isolated lineage, suggesting local host adaption to Myotis sodalis (the Indiana bat). Previous studies have shown that P. destructans displays extreme sensitivity to UV-C due to a lack of a *UVE1* homologue, a key component of the UV light repair pathway (21). The exploitation of genes and mechanisms identified via comparative genomics that are leading to adaptation and survival of P. destructans could eventually prove crucial for the management of P. destructans disease.

Since there is no bat migration between Europe and North America, it is reasonable to assume that human activity is responsible for the introduction of P. destructans into North America (7, 22). The results of the study by Drees et al. demonstrate that even when suffering a strong genetic bottleneck, P. destructans can successfully establish within new ecosystems in naïve hosts. With the increase in the number of EFPs affecting wildlife populations over recent decades (2), it is clear we are facing a conservation crisis for species vital to the healthy functioning of our ecosystems. As with other fungal epizootic infections, there is great need for a tightening of global biosecurity (2).
